# QuEChERS-同位素稀释-气相色谱-串联质谱法测定动物源性食品中9种*N*-亚硝胺类化合物

**DOI:** 10.3724/SP.J.1123.2020.06010

**Published:** 2021-01-08

**Authors:** Xiangyi KONG, Lili ZHUANG, Enhua FANG, Peng LIN, Zilong ZHENG, Xianghua ZHENG, Dunming XU

**Affiliations:** 1.集美大学水产学院, 福建 厦门 361021; 1. Fisheries College of Jimei University, Xiamen 361021, China; 2.厦门海关技术中心, 福建 厦门 361000; 2. Xiamen Customs Technology Center, Xiamen 361000, China; 3.福建省市场监督管理局, 福建 福州 350003; 3. Fujian Administration for Market Regulation, Fuzhou 350003, China

**Keywords:** 气相色谱-串联质谱, QuEChERS, *N*-亚硝胺类化合物, 动物源性食品, gas chromatography-tandem mass spectrometry (GC-MS/MS), QuEChERS, *N*-nitrosamines, animal derived foods

## Abstract

建立了同时测定动物源性食品中9种*N*-亚硝胺类化合物的气相色谱-串联质谱分析方法。当下动物源性食品中*N*-亚硝胺类化合物污染种类较多,对人体危害较大,但国标GB 5009.26-2016仅针对*N*-二甲基亚硝胺的检测,且存在样品前处理复杂、标准方法回收率低、再现性差等问题,因此建立同时快速检测多种*N*-亚硝胺类化合物的方法有一定现实意义。称取10.0 g样品,置于50 mL离心管中,加入200 μL内标工作液和10 mL乙腈,冷冻30 min后,加入4 g硫酸镁和1 g氯化钠进行脱水,以9000 r/min离心5 min。取5 mL上清液使用150 mg聚苯乙烯二乙烯苯(PLS-A)粉末净化,再使用1.6 g MgSO_4_和0.4 g NaCl脱水,过0.22 μm滤膜,上机分析。在初始温度为50 ℃时采用程序升温模式,0.16 min后,以900 ℃/min的速率将温度升至220 ℃。采用毛细管气相色谱柱HP-Innowax(30 m×0.25 mm×0.25 μm)分离,使用电子轰击电离(EI)源检测,在多反应监测模式下,以保留时间和特征离子对信息进行定性和定量分析,使用内标法定量*N*-亚硝胺类化合物。结果表明,*N*-亚硝胺类化合物在0.1~50.0 μg/L范围内具有良好的线性关系,方法的检出限(*S/N*=3)和定量限(*S/N*=10)分别为0.03~0.30 μg/kg和0.10~1.00 μg/kg。对不同样品基质进行0.5、1.0、3.0 μg/kg3个水平的加标回收试验,9种*N*-亚硝胺类化合物的回收率为80.4%~98.5%, RSD(*n*=6)为2.41%~12.50%。应用建立的方法检测市面上常见的动物源性食品,除*N*-亚硝基乙胺、*N*-亚硝基吗啡胆碱外,其他7种*N*-亚硝胺类化合物均有不同程度检出。检测结果表明,腌制水产品中*N*-亚硝胺类化合物含量普遍高于其他样品。研究建立的方法操作简单,不需要长时间蒸馏提取,可快速对动物源性食品中*N*-亚硝胺类化合物进行定性和定量分析,且样品和试剂的消耗量更少,节省成本,对环境污染小。该法的建立对我国动物源性食品中*N*-亚硝胺类化合物残留水平的控制、检测标准的制定和采取相应的管理措施具有一定的理论和现实意义。

## Abstract (en)

In this study, a comprehensive analytical method based on gas chromatography-tandem mass spectrometry (GC-MS/MS) was developed for the determination of nine *N*-nitrosamines in animal derived foods. There are many kinds of *N*-nitrosamines in foods that are harmful to human health. However, the national standard GB 5009.26-2016 pertains only to the detection of *N*-dimethylnitrosamine; there are many drawbacks of this method, such as complicated sample preparation, low recovery rate, and poor reproducibility. Hence, it is of practical significance to establish a method for the simultaneous determination of a variety of *N*-nitrosamines. The optimal extraction conditions for the developed method were as follows: 10.0 g aliquots of the sample were placed in a 50 mL centrifuge tube, followed by the addition of 10 mL acetonitrile and 200 μL internal working standard solutions. After 30 min of freezing treatment, 4 g magnesium sulfate and 1 g sodium chloride were added for dehydration, and the tube was centrifuged at 9000 r/min for 5 min. After vortex centrifugation, 5 mL of the clear supernatant was purified using 150 mg polystyrene divinylbenzene (PLS-A). The purified extracts were dewatered using 1.6 g MgSO_4_ and 0.4 g NaCl, and then filtered through a 0.22 μm membrane filter unit prior to GC-MS/MS analysis. Temperature-programmed was applied at an initial temperature of 50 ℃. After 0.16 min, the temperature was raised to 220 ℃ at the rate of 900 ℃/min for 5 min. *N*-Nitrosamines were separated on an HP-Innowax column (30 m×0.25 mm×0.25 μm). Identification and quantification were achieved using an electron impact ion (EI) source in positive ion mode with multiple reaction monitoring (MRM). The internal standard method was used to quantify the *N*-nitrosamines. Under the optimal conditions, the correlation coefficients of the standard calibration curves were not less than 0.99 in the range of 0.1-50.0 μg/L. The limits of detection were 0.03-0.30 μg/kg (*S/N*=3), and limits of quantification were 0.15-1.00 μg/kg (*S/N*=10). At spiked levels of 0.5, 1.0, and 3.0 μg/kg, the average recoveries of *N*-nitrosamines in spiked samples ranged from 80.4% to 98.5%, with relative standard deviations between 2.41% and 12.50%. This method was used to determine animal derived food products, except *N*-itrosomethylethylamine and *N*-nitrosomorpholine, others were founded. The results showed that *N*-nitrosamines levels in salted aquatic products were generally higher than those of the other samples. The method established in this study is simple to operate, and it does not require any time-consuming distillation extraction. Furthermore, there is minimal consumption of samples and reagents; consequently, the experiment cost is reduced, and the method is environmentally friendly. This method has theoretical and practical significance for the control of *N*-nitrosamines residues in animal derived foods, establishment of detection standards, and corresponding management measures.

亚硝胺是一类含有*N*-亚硝基(*N*-NO)的亚硝基化合物^[1]^,广泛存在于饮用水、熏肉制品和腌制蔬菜中^[2]^。蛋白质腐败分解时可以产生胺类物质,因此蛋白质含量丰富的食物往往容易*N*-亚硝胺类化合物含量超标,此外经过烟熏、油炸、腌制的动物源性食品也容易产生亚硝胺^[3]^。1937年,Freund^[4]^第一次报道了两例由于职业接触*N*-亚硝基二甲胺(NDMA)而导致中毒的案例,目前已经证实亚硝胺对生物具有很强的基因毒性,可诱导人体的食管、肝脏和肾脏等器官发生癌症^[5]^,其中NDMA、*N*-二乙基亚硝胺(NDEA)被国际癌症研究机构(IRAC)评定为2A级致癌物,其他的*N*-亚硝胺类化合物为2B级致癌物。目前,我国在GB 2762-2017食品安全国家标准 食品中污染物限量》中也对动物源性食品中NDMA限量指标做了要求,其中肉及肉制品(肉类罐头除外)的限量为3 μg/kg,水产动物及其制品(水产品罐头除外)的限量为4 μg/kg。动物源性食品中的*N*-亚硝胺类化合物严重危害人体健康,如何控制动物源性食品生产过程中*N*-亚硝胺类化合物的产生也是当前*N*-亚硝胺类化合物研究的重要方向,开发对*N*-亚硝胺类化合物含量进行快速检测和定量的技术尤为重要^[6]^。

热能分析仪作为基于化学发光设计的专门测定*N*-亚硝胺类化合物的检测器,具有检测快速、灵敏度高的优点^[7,8]^,但其价格昂贵,应用范围窄,且使用过程中经常需要检修和维护,一般实验室都未配备,故基于热能分析仪的*N*-亚硝胺类化合物检测法未能广泛应用。此外,针对亚硝胺的检测方法还有色谱-核磁法^[9]^、电化学检测法^[10,11,12,13]^、液相色谱法^[14]^、液相色谱-质谱法^[15,16]^、气相色谱-氮磷检测器法^[17]^、气相色谱-质谱法^[18,19,20]^等。其中电化学检测方法的精度和灵敏度仍有待提高;液相色谱-串联质谱法对相对分子质量较小的*N*-亚硝胺类化合物的响应无法达到要求;气相色谱-质谱法容易产生NDMA假阳性现象,气相色谱-串联质谱法因其高特异性和高灵敏度,是目前针对*N*-亚硝胺类化合物检测最为广泛使用的方法。

*N*-亚硝胺类化合物的前处理方法主要包括液液萃取法、水蒸气蒸馏萃取法、超临界萃取法和QuEChERS等。目前国内对*N*-亚硝胺类化合物的标准检测方法为GB 5009.26-2016《食品安全国家标准 食品中*N*-亚硝胺类化合物的测定》,其提取方法为水蒸气蒸馏法,但该方法的样品需求量过大,操作过程复杂,耗时较长,且回收率波动较大;朱萌萌等^[21]^采用蒸馏萃取法结合气相色谱-串联质谱法对肉制品中10种挥发性*N*-亚硝胺类化合物进行检测,可实现蒸馏和提取同时进行,但仍存在耗时较长和样品用量大等问题,在面对大量待测样品时具有一定的局限性;何淑娟等^[22]^和高蕙文等^[23]^利用净化剂对*N*-亚硝胺类化合物提取液进行净化后氮吹至近干,但*N*-亚硝胺类化合物在氮吹至近干时损失较大,容易造成回收率较低;李玮等^[24]^使用*N*-丙基乙二胺(PSA)、十八烷基键合硅胶(C18)和无水硫酸钠对*N*-亚硝胺类化合物提取液进行净化和除水,但PSA的净化效果较弱,可能导致定量结果不准确;硫酸钠除水效果较弱,可能造成除水不彻底,从而影响仪器性能。

QuEChERS是近年来国际上兴起的一种新型农产品检测的快速样品前处理技术,是利用基质分散萃取机理来吸附样品中的杂质,从而达到保留目标物和净化样品的目的。本文通过优化样品前处理条件、色谱和质谱条件,建立了QuEChERS-同位素稀释-气相色谱-串联质谱同时快速测定动物源性食品中9种*N*-亚硝胺类化合物的方法。该方法具有操作简单、提取高效、更加经济的优点,可准确、快速测定动物源性食品中的*N*-亚硝胺类化合物。

## 1 实验部分

### 1.1 仪器与试剂

7890气相色谱-7000三重四极杆质谱仪(美国Agilent公司); 2-16KL高速离心机(德国Sigma公司); VORTEX 3涡旋混匀仪(德国IKA公司)。

乙腈、二氯甲烷、乙酸乙酯均为色谱纯(德国Merck公司); MgSO_4_和NaCl均为分析纯(广州西陇化工有限公司),聚苯乙烯二乙烯苯聚合物(PLS-A)、十八烷基键合硅胶吸附剂(C18)(北京迪马公司);增强型脂质去除(EMR)小柱(美国Agilent公司);实验用水由Milli-Q超纯水系统(美国Millipore公司)制备。

9种*N*-亚硝胺类化合物标准品包括NDMA、*N*-亚硝基乙基甲基胺(NMEA)、NDEA、*N*-亚硝基二丁胺(NDBA)、*N*-亚硝基二丙胺(NDPA)、*N*-亚硝基哌啶(NPIP)、*N*-亚硝基吗啉(NMorPh)、*N*-亚硝基二苯胺(NDPhA)及内标*N*-亚硝基二甲胺-d6(NDMA-d6)、*N*-亚硝基二正丙胺-d14(NDPA-d14),均购自英国LGC公司,质量浓度均为1000 μg/mL。

### 1.2 实验方法

1.2.1 标准溶液的配制

将9种*N*-亚硝胺类化合物标准品(1000 μg/mL)用乙腈稀释配制成1.0 μg/mL的混合标准工作液,低于-18 ℃避光保存,有效期6个月。

将内标标准品(1000 μg/mL)用乙腈稀释配制成1.0 μg/mL的内标工作液,低于-18 ℃避光保存,有效期6个月。

1.2.2 样品前处理

液态样品摇匀后提取处理;粉状样品直接提取处理;其他样品取可食部分组织捣碎。将制备好的试样于0~5 ℃冷藏保存,待测。

提取:称取10 g(精确至0.01 g)试样,置于50 mL离心管中,加入200 μL内标工作液和10 mL乙腈,涡旋1 min混匀,置于冰箱-20 ℃冷冻30 min,加入陶瓷均质子2粒、4 g MgSO_4_和1 g NaCl,涡旋1 min,于0 ℃以9000 r/min离心5 min,上清液待净化。

净化:取150 mg PLS-A粉末和5 mL水,置于15 mL离心管中,振荡后加入5 mL上述上清液,并涡旋1 min,于0 ℃以9000 r/min离心5 min。

除水:将净化液转移至另一15 mL离心管中,加入1.6 g MgSO_4_和0.4 g NaCl,涡旋30 s,于0 ℃以9000 r/min离心5 min,取1 mL上层有机相过0.22 μm微孔滤膜,然后移入进样瓶中上机检测。

1.2.3 分析条件

色谱柱:毛细管气相色谱柱HP-Innowax(30 m×0.25 mm×0.25 μm,美国Agilent公司);进样口温度:220 ℃;载气:氦气;流速60 mL/min;溶剂放空模式;程序升温模式:初始温度50 ℃,保持0.16 min,以900 ℃/min升温至220 ℃,保持5 min;进样量5 μL。

电子轰击电离(EI)源;离子源温度250 ℃;传输线温度250 ℃;溶剂延迟6 min;电子能量:70 eV;采集模式:MRM模式。9种亚硝胺类化合物及内标的保留时间、前体离子、子离子、碰撞能量(CE)见表1。

**表 1:** 9种*N*-亚硝胺类化合物及2种内标的质谱参数

Compound	CAS No.	Retention time/min	Precursor ion (m/z)	Product ions (m/z)	Collision energies/eV	IS
N-Nitrosodimethylamine (NDMA)	62-75-9	6.29	74.0	42.1, 44.0^*^	19, 3	NDMA-d6
N-Nitrosodimethylamine-d6 (NDMA-d6)	17829-05-9	6.28	80.0	46.1, 50.1^*^	22, 5	-
N-Nitrosomethylethylamine (NMEA)	10595-95-6	6.65	88.0	42.0, 71.0^*^	17, 2	NDMA-d6
N-Nitrosodiethylamine (NDEA)	55-18-5	6.88	102.0	56.0, 85.0^*^	15, 2	NDMA-d6
N-Nitrosodi-n-propylamine (NDPA)	621-64-7	7.98	130.2	43.0, 113.1^*^	9, 7	NDPA-d14
N-Nitrosodi-n-propylamine-d14 (NDPA-d14)	93951-96-3	7.93	144.1	50.1, 126.1^*^	11, 1	-
N-Nitrosodi-n-butylamine (NDBA)	924-16-3	9.44	116.0	74.1, 99.0^*^	8, 2	NDPA-d14
N-Nitrosopiperidine (NPIP)	100-75-4	9.73	114.0	41.0, 84.0^*^	13, 5	NDPA-d14
N-Nitrosopyrrolidine (NPYR)	930-55-2	9.98	100.0	43.1, 55.1^*^	9, 5	NDPA-d14
N-Nitrosomorpholine (NMorPh)	59-89-2	10.37	116.2	56.0, 86.1^*^	11, 5	NDPA-d14
N-Nitrosodiphenylamine (NDPhA)	86-30-6	14.16	169.0	167.1, 168.1^*^	30, 17	NDPA-d14

^*^Quantitative ion.

## 2 结果与讨论

### 2.1 提取液的选择

*N*-亚硝胺类化合物相对分子质量较小,极性较强,在强极性溶剂中溶解性更强,常用的提取溶剂有乙腈、二氯甲烷和乙酸乙酯^[25]^。实验对这3种溶剂进行对比,结果见图1。可以看出,乙腈对*N*-亚硝胺类化合物的溶解性较强,对油脂和色素等杂质溶解度相对较小,提取效率最佳;乙酸乙酯对极性强的*N*-亚硝胺类化合物提取效果较差,且容易与油脂共萃取,从而对上机造成干扰;二氯甲烷提取效果差,且操作中由于挥发性极强容易造成误差。因此本实验选择乙腈作为提取溶剂。

**图 1:**
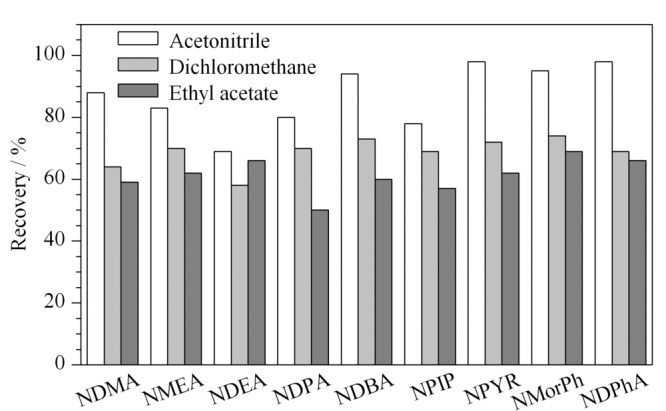
不同提取溶剂对9种*N*-亚硝胺类化合物回收率的影响

### 2.2 吸附剂的选择

肉制品、水产品等动物源性食品通常含有色素、脂肪酸等复杂基质,很容易影响测定的准确性。C18通常用来去除脂肪酸、色素,同时吸附非极性化合物,PLS-A可较好地吸附有机酸、脂肪酸、糖、色素等干扰物质。根据何淑娟等^[22]^的研究,本文选取PLS-A粉末、C18粉末和EMR小柱对其净化效果进行对比。准备待测样品3份,将样品中加入200 μL *N*-亚硝胺类化合物内标工作液,其中两份样品分别用150 mg PLS-A和150 mg C18进行净化处理,第三份样品采用EMR小柱净化,3份样品按照1.2.2节进行处理,其中EMR小柱加入1 mL水进行活化后再加入1.2.2节的待净化液4 mL进行净化,样品回收率见图2。结果表明,采用PLS-A时,净化效果较好,对油脂、色素等杂质的净化效果理想。因此本文采用PLS-A作为净化剂。

**图 2:**
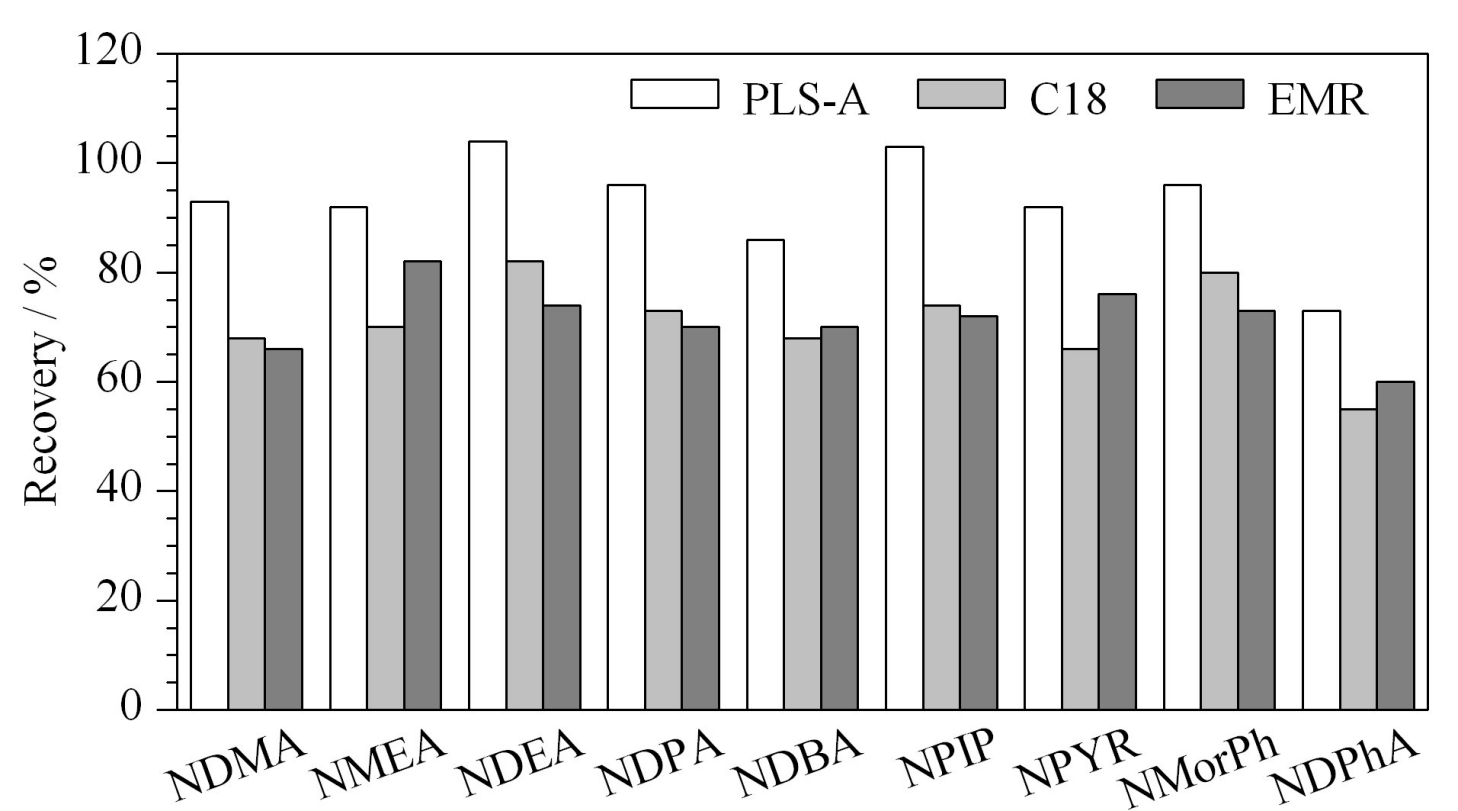
不同净化方式对9种*N*-亚硝胺类化合物回收率的影响

经由PLS-A净化后的溶液含水,需除水后才能进行仪器分析,本方法采用MgSO_4_和NaCl作为除水剂。NaCl可以增强溶液极性,减小*N*-亚硝胺类化合物在水中的溶解度,得到更高的萃取效率;MgSO_4_除水能力更好,但在吸水过程中会大量放热,亚硝胺在高温条件下易挥发,减少MgSO_4_的用量可减少损失,但减少过多又容易导致除水不彻底。经对比研究最终采用1.6 g MgSO_4_和0.4 g NaCl除水。

### 2.3 色谱、质谱条件的选择

参考翟孟婷等^[26]^的研究结果,选用毛细管气相色谱柱HP-Innowax (30 m×0.25 mm×0.25 μm)。按1.2.3节条件对9种*N*-亚硝胺类化合物进行测定,得到总离子流色谱图和MRM色谱图(见图3)。

**图 3:**
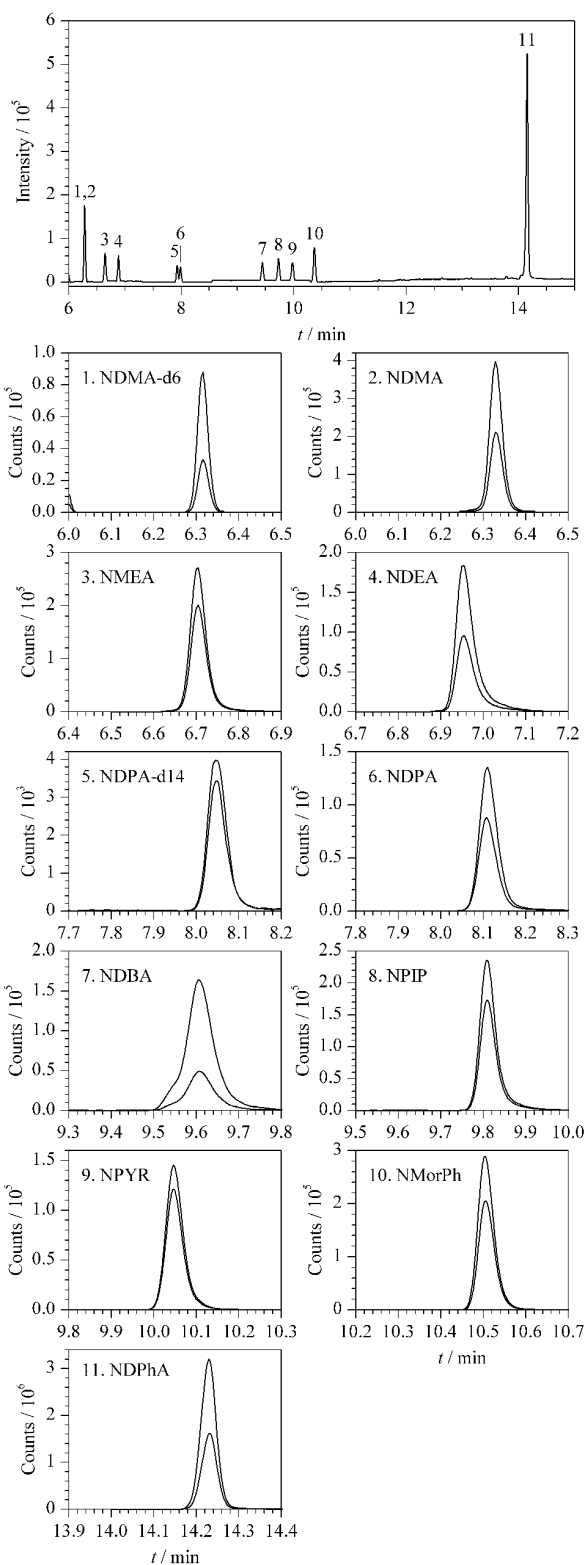
9种*N*-亚硝胺类化合物及其内标的总离子流和MRM色谱图(10 μg/L)

### 2.4 基质效应

基质效应(ME)是指在测定过程中,由于待测成分的离子化效应被改变,从而信号受到增强或者抑制的现象。基质效应会影响结果的准确度。本文参考Al-Kaseem等^[14]^的方法,采用标准曲线法(基质效应=基质匹配溶液斜率与溶剂标准溶液斜率的比值×100%)来考察*N*-亚硝胺类化合物在动物源性食品中的基质效应。ME值在85%~115%之间可认为该基质不存在基质效应。用空白样品提取液作为标准溶液的稀释溶液,使标准溶液和样品溶液的离子化效应相同。结果表明,9种*N*-亚硝胺类化合物的ME值均小于70%,可认为亚硝胺在动物源性食品中表现出较强的基质抑制效应,为确保测定的准确性,本文采用内标法进行准确定量。

### 2.5 标准曲线、检出限和定量限

精密吸取9种*N*-亚硝胺类化合物混合标准工作液和内标工作溶液适量,用乙腈稀释成质量浓度为0.1、0.5、1.0、5.0、20.0和50.0 μg/L的系列混合标准溶液,其中内标的质量浓度均为20.0 ng/mL,实验按浓度由低到高的顺序进样分析。以*N*-亚硝胺类化合物及其对应氘代同位素内标的浓度比值为横坐标,以*N*-亚硝胺类化合物及其对应氘代同位素内标的峰面积比值为纵坐标,绘制标准曲线,从而获得线性方程和相关系数(*R*^2^),见表2。结果表明,线性相关系数均不小于0.99,说明*N*-亚硝胺类化合物在0.1~50.0 μg/L范围内有良好的线性关系。以定量离子信噪比(*S/N*)为3和10时的响应定义方法的检出限(LOD)和定量限(LOQ), 9种*N*-亚硝胺类化合物的检出限和定量限分别为0.03~0.30 μg/kg和0.10~1.00 μg/kg。

**表 2:** 9种*N*-亚硝胺类化合物的线性方程、相关系数、检出限和定量限

Compound	Linear equation	R^2^	LOD/ (μg/kg)	LOQ/ (μg/kg)
NDMA	y=0.96x+0.04	0.997	0.05	0.15
NMEA	y=0.87x+0.04	0.996	0.10	0.30
NDEA	y=0.68x+0.03	0.997	0.10	0.30
NDPA	y=1.57x+0.08	0.996	0.10	0.30
NDBA	y=2.76x+0.23	0.990	0.10	0.30
NPIP	y=2.92x+0.14	0.996	0.30	1.00
NPYR	y=1.80x+0.08	0.996	0.20	0.60
NMorPh	y=3.72x+0.19	0.996	0.10	0.30
NDPhA	y=37.57x+1.68	0.997	0.03	0.10

*y*: peak area ratio of the quantitative ion of the analyte to the internal standard; *x*: mass concentration ratio of the analyte to the internal standard.

### 2.6 准确度和精密度

按照前述方法,对不含*N*-亚硝胺类化合物的鱼肉、虾肉、牛肉和腊肠等4种空白样品进行添加回收试验,设定添加水平为0.5、1.0、3.0 μg/kg,考察方法的准确度和精密度,结果见表3。结果表明,9种*N*-亚硝胺类化合物的回收率为80.4%~98.5%, RSD为2.41%~12.50%,方法准确度和精密度良好。

**表 3:** 9种*N*-亚硝胺类化合物在动物源性食品中的添加回收率和相对标准偏差(*n*=6)

Analyte	Spiked/ (μg/kg)	Fish			Shrimp			Beef			Preserved pork		
		Recovery/%	RSD/%		Recovery/%	RSD/%		Recovery/%	RSD/%		Recovery/%		RSD/%
NDMA	0.5	85.9	9.58		88.5	10.80		86.5	11.80		84.1		10.80
	1.0	85.3	3.51		96.3	8.51		84.3	7.51		82.3		6.51
	3.0	97.1	3.10		92.0	5.14		91.1	3.14		95.1		2.41
NMEA	0.5	86.5	10.20		86.5	11.50		82.4	8.11		89.5		12.50
	1.0	92.5	4.12		91.2	7.85		92.0	4.55		90.1		5.11
	3.0	96.4	2.85		97.3	2.45		96.0	2.82		95.4		2.74
NDEA	0.5	89.7	10.70		89.5	9.23		83.5	11.60		84.1		11.40
	1.0	90.4	3.57		92.4	6.88		94.1	5.98		93.1		3.78
	3.0	97.6	2.77		96.5	2.85		98.5	2.96		97.5		2.78
NDPA	0.5	91.4	11.10		88.5	9.44		89.8	9.04		85.2		11.20
	1.0	94.1	7.45		95.2	8.55		96.3	8.87		94.2		8.51
	3.0	98.4	3.74		98.5	3.25		97.2	3.71		97.2		3.28
NDBA	0.5	90.7	9.85		82.1	8.51		83.7	7.61		87.2		10.20
	1.0	92.8	7.56		91.4	7.44		92.8	7.74		91.4		7.94
	3.0	97.5	2.88		97.1	4.12		98.5	3.52		96.5		3.87
Analyte	Spiked/ (μg/kg)	Fish			Shrimp			Beef			Preserved pork		
		Recovery/%	RSD/%		Recovery/%	RSD/%		Recovery/%	RSD/%		Recovery/%		RSD/%
NPIP	0.5	94.5	8.57		81.1	9.21		84.5	8.11		92.5		9.18
	1.0	93.4	8.35		92.8	6.89		97.1	7.05		95.2		9.00
	3.0	96.3	3.04		95.6	3.57		96.3	3.54		95.4		3.95
NPYR	0.5	82.5	9.88		85.2	9.77		89.7	12.40		96.5		9.04
	1.0	92.7	8.57		89.5	6.22		90.5	8.95		92.5		8.44
	3.0	97.4	3.98		95.6	5.47		96.5	3.57		95.2		4.12
NMorPh	0.5	85.5	8.35		88.5	9.02		82.8	8.93		95.4		8.21
	1.0	89.3	6.54		89.2	4.58		89.5	5.23		86.8		5.89
	3.0	95.3	3.52		94.8	3.28		95.1	4.57		90.4		3.75
NDPhA	0.5	80.4	8.04		84.3	7.81		88.5	6.83		95.4		7.98
	1.0	92.0	3.47		86.0	5.14		89.7	6.52		85.8		7.14
	3.0	98.1	3.07		94.5	2.58		97.1	2.56		85.4		2.71

### 2.7 实际样品检测

按照本文建立的方法对采集的60批动物源性食品,包括肉糜、腌制肉制品、水产制品等样品进行分析,有18批样品中检出*N*-亚硝胺类化合物(见表4),其中NDMA、NDBA、NPIP、NPYR、NDPhA的检出率最高,且部分腌制水产品的NDMA含量高出我国GB 2762-2017《食品安全国家标准 食品中污染物限量》中的限量值(NDMA≤3 μg/kg),其中腌制鱿鱼丝样品中的NDMA含量甚至高达7.93 μg/kg。

**表 4:** 实际样品中9种*N*-亚硝胺类化合物的检测结果

Compound	Contents/(μg/kg)					
	Meat paste		Pickled bacon		Aquatic products	
NDMA	0.05-	1.13	0.70-	4.65	0.30-	7.93
NMEA	N	D	N	D	N	D
NDEA	0-	2.42	0-	3.27	0.10-	2.55
NDPA	0.12-	1.41	0.03-	0.88	0-	1.33
NDBA	0-	1.72	0-	1.65	0-	3.16
NPIP	0-	1.06	0-	1.95	0-	1.84
NPYR	0.45-	2.42	0-	1.35	0-	4.38
NMorPh	N	D	N	D	N	D
NDPhA	0-	2.01	0-	1.84	0-	2.01

ND: not detected.

## 3 结论

建立了QuEChERS-同位素稀释-GC-MS/MS测定动物源性食品中9种*N*-亚硝胺类化合物残留的方法。方法的回收率和精密度良好,线性范围广,重复性好,检出限低,可以同时对9种*N*-亚硝胺类化合物快速地进行定性和定量分析,从而为动物源性食品中*N*-亚硝胺类化合物含量的安全评价提供依据。
